# Reproductive success of jack and full-size males in a wild coho salmon population

**DOI:** 10.1098/rsos.221271

**Published:** 2023-04-05

**Authors:** Erika M. King, David A. Tallmon, Scott C. Vulstek, Joshua R. Russell, Megan V. McPhee

**Affiliations:** ^1^ College of Fisheries and Ocean Sciences, University of Alaska, 17101 Point Lena Loop Road, Juneau, AK 99801, USA; ^2^ Biology and Marine Biology Program, University of Alaska Southeast, 11066 Auke Lake Way, Juneau, AK 99801, USA; ^3^ National Oceanic and Atmospheric Administration, 17109 Point Lena Loop Road, Juneau, AK 99801, USA

**Keywords:** life history, mating systems, reproductive success

## Abstract

Despite the wealth of research on Pacific salmon *Oncorhynchus* spp. life histories there is limited understanding of the lifetime reproductive success of males that spend less time at sea and mature at a smaller size (jacks) than full-size males. Over half of returning male spawners can be jacks in some populations, so it is crucial to understand their contribution to population productivity. We quantified adult-to-adult reproductive success (RS) of jacks and their relative reproductive success (RRS) compared to full-size males in a wild population of coho salmon in the Auke Creek watershed, Juneau, Alaska. We used genetic data from nearly all individuals (approx. 8000) returning to spawn over a decade (2009–2019) to conduct parentage analysis and calculate individual RS. The average adult-to-adult RS of jacks (mean = 0.7 and s.e. = 0.1) was less than that of full-size males (mean = 1.1 and s.e. = 0.1). Jack RRS was consistently below 1.0 but ranged widely (0.23 to 0.96). Despite their lower average success, jacks contributed substantially to the population by siring 23% of the total returning adult offspring (1033 of 4456) produced between 2009 and 2015. Our results imply that jacks can affect evolutionary and population dynamics, and are relevant to the conservation and management of Pacific salmon.

## 1. Introduction

In Pacific salmon *Oncorhynchus* spp., extensive variation in life-history strategies and traits has enabled their wide-ranging distribution and persistence for millions of years. Diversity in life-history types is beneficial for population resilience [1–3] and different life histories can result in dissimilar reproductive success (RS). Lifetime RS of individuals influences trends in population size which affect levels of inbreeding and genetic drift and ultimately the capacity of the population to adapt [4]. Therefore, understanding lifetime RS and the contribution of different life histories within populations is a prerequisite for developing effective conservation strategies and assessing whether current management programs are successful.

The balance between survival and achieving reproduction amid intense male competition in Pacific salmon [5] has contributed to the presence of distinct life-history types representing variation in age and size at maturity. Males exhibit multiple life-history tactics related to these traits. Sockeye salmon *Oncorhynchus nerka*, chinook salmon *Oncorhynchus tshawytscha,* and coho salmon *Oncorhynchus kisutch* exhibit alternate male life-history forms: full-size males and jacks. Full-size males typically maintain access to females by fighting and guarding. Jacks, on the other hand, spend less time at sea and mature at a smaller size than full-size males and females, and achieve spawning success by taking up satellite positions around the female then sneaking in to release their sperm as the eggs are being laid [6] instead of fighting. The term ‘jack’ refers to anadromous males and is different from ‘precocial male’ which typically refers to males that sexually mature as parr without migrating to sea (also known as mature male parr), as seen in some populations of chinook salmon, steelhead trout and Atlantic salmon.

Early male maturation (which refers to jacks and mature male parr) is influenced by both environmental and genetic factors, but these factors are difficult to parse out. Males that mature early usually reach the critical maturation threshold of size, growth rate and energy stored sooner than full size males [7,8]. Evidence for the genetic component of early male maturation in Pacific salmon comes from studies that show: (i) families sired by jacks have higher rates of jacks [9,10]; (ii) moderate to high heritability (0.49–0.54) of age at maturity ([11]; see also [10]); and (iii) genomic regions and haplotypes associated with age at maturity [12,13]. Interestingly, the genetic regulation of age at maturity may be different for jacks than precocial male parr [14,15]. Early male maturation has also been linked with size obtained in freshwater [16] and hatchery release date and size [17,18], indicating that environmental factors can influence the rate of early male maturation, particularly in high-growth environments such as hatcheries [19,20]. Frequency-dependent disruptive selection is hypothesized to maintain the early male maturation life-history tactic in the wild [6,21]. Jacks and mature male parr are thought to be more successful at sneaking when they are rare owing to reduced competition for sneaking positions and because full-size males are focused primarily on battling with other full-size males [6]. However, a status-dependent conditional strategy [22,23] could also explain the maintenance of early male maturation in a population even if they have lower mean fitness than regular males.

Numerous observational and experimental genetic studies document the RS of jacks. The proportion of eggs fertilized by jacks ranges widely but can be substantial. One study of sockeye salmon found that the percentage of eggs fertilized by jacks on an individual basis ranged from 3 to 93% [24]. A study of chinook salmon found that jacks cumulatively sired 20% of total offspring at the fry stage (population level production) [25]. In cases when adult-to-adult RS estimates are not available it is possible to use adult-to-juvenile estimates [26], although the impact of all factors after the juvenile stage are not included. Therefore, adult-to-adult RS estimates are highly valuable, as they provide a more direct measure of Darwinian fitness [27–29].

It is important to understand how individuals with alternative reproductive strategies contribute to population viability and under what conditions. This is relevant for managing wild populations because the contribution of jacks is often ignored: their abundance is not always monitored and when it is, they are prone to being undercounted [30]. The adult-to-adult RS of jacks is rarely quantified in natural systems (but see [31]), and their RS relative to full-size males is not well understood. Studies that have analysed the RS of hatchery jacks and full-size males separately (adult-juvenile RS, [32]; adult-adult RS, [33–35]) have found that jacks typically have lower RS than full-size hatchery-origin males [36]. Insight on natural age at maturity and mating structure has implications for hatcheries, as it would inform how captive breeding methods differ from wild mating systems. In hatcheries that attempt to mate pairs randomly but exclude jacks, mating is selective. The impact of this divergence from natural mating structure is unknown.

We sought to improve understanding of natural mating structure and the jack life-history tactic by quantifying jack RS and relative reproductive success (RRS) of jacks compared to full-size males in a naturally spawning population of coho salmon in Auke Creek, Juneau, Alaska. The access to every individual returning to Auke Creek provided a unique opportunity to study an important life-history tactic that has implications for conservation and management. We defined individual RS as the number of offspring that survive to adulthood, which encompasses that individual's mating success, fecundity and the probability of the offspring's survival. Here we show that coho salmon jacks can contribute a substantial proportion of returning adult offspring to the population, even if their individual reproductive success is lower on average than that of full-size males.

## 2. Material and methods

### 2.1. Study population

**Figure 1.**
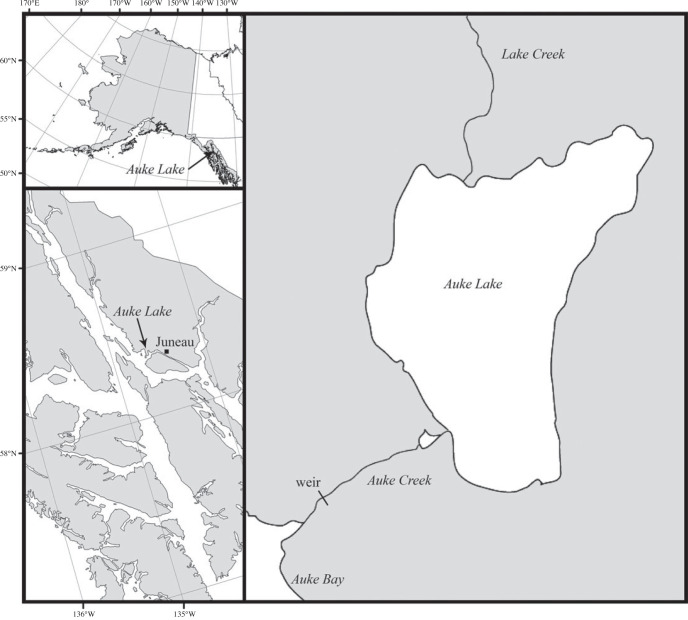
Map of Auke Lake system in southeastern Alaska near Juneau. Auke Creek weir is located between Auke Lake and Auke Bay.

**Figure 2.**
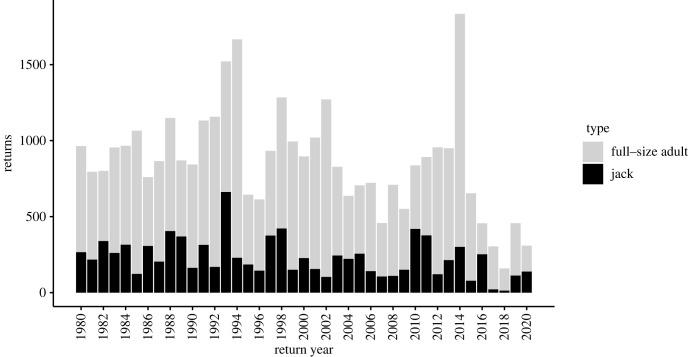
Number of returning full-size (male and female) and jack coho salmon counted at the Auke Creek weir from 1980 to 2018.

The study was conducted in the Auke Lake drainage, Juneau, Alaska (figure 1), which supports populations of sockeye salmon, pink salmon *Oncorhynchus gorbuscha* and coho salmon, as well as cutthroat trout *Oncorhynchus clarkii*, Dolly Varden *Salvelinus malma*, coastrange sculpin *Cottus aleuticus* and very few rainbow trout *Oncorhynchus mykiss*. The National Oceanic and Atmospheric Administration (NOAA) operates a weir capable of capturing both emigrant and immigrant salmonids in the lake outlet, Auke Creek. The abundance of coho salmon smolts and returning adults (figure 2) has been recorded every year since 1980. Each immigrating adult salmon is transported over the weir manually, allowing every returning fish to be sampled. Since 2009, all returning adult coho salmon have been sampled for genetic analysis: an axillary process is removed and stored in 95% ethanol for later genotyping. Of the returning adult fish, roughly 33% are sampled for age, sex, and length. Age is determined using scales from the preferred area by NOAA personnel following the methods outlined by [37]. Fish are identified as female, male, or jack based on external morphological characteristics. Jacks have the same snout, head, and body shape as full-size males, but are easily distinguishable from full-size males because they do not overlap in size distribution (S. Vulstek (NOAA) 2019, personal communication; electronic supplementary material, figure S1), and females are distinguished from full-size males based on head shape, vent size and shape, and overall body shape. Because all juvenile coho salmon emigrating from Auke Lake are coded wire tagged and given an adipose fin clip mark, any returning adult possessing an intact adipose fin is identified as a putative stray (i.e. immigrant).

**Figure 3.**
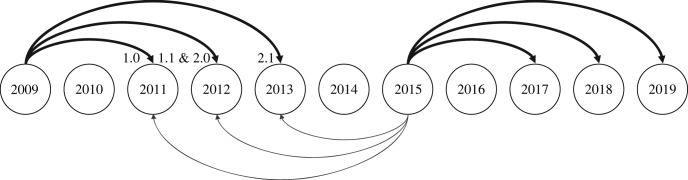
Auke Creek coho salmon life-history diagram. Circles indicate return years (2009–2019). Individuals in this population return 2–4 years after being spawned. Thick black lines relate a single brood (parent) year to all possible offspring return years. A thin grey line relates a single return year to all possible brood years. The number next to each circle indicate the age of the fish. The number before the decimal place is the years spent in freshwater and the number after the decimal point is the years spent in the ocean. For example: 1.0 indicates a fish that spent one year in freshwater and less than a year in the ocean.

This project used demographic data and tissues from nearly all adult coho salmon that returned to the weir from 2009 to 2019. We focused on brood years 2010, 2012, 2013 and 2014, which were the years with the highest ratio of jacks, lowest ratio of jacks, median ratio of jacks and highest number of spawners, respectively. Auke Creek coho salmon spend one or two years in freshwater and either six months (jacks) or a full year (adult females and full-size males) at sea before returning to spawn (figure 3). Given the two options for time spent in freshwater, it is possible for jacks and full-size males to return to spawn at the same age. For example, a jack that spent two years in freshwater and six months at sea would return at the same age as a full-size male that spent one year in freshwater and one year at sea. All analyses were performed using R Statistical Software (v. 4.0.3) [38].

### 2.2. Genotyping

All returning adult coho salmon from 2009 to 2019 were genotyped. Tissue samples were sent to GTseek (https://gtseek.com/) for Chelex DNA extraction, library preparation, and amplicon sequencing using the ‘genotyping-in-thousands by sequencing’ protocol [39]. The samples were genotyped at a panel of 259 single-nucleotide polymorphism (SNP) loci developed by the Columbia River Inter-Tribal Fisheries Commission for coho salmon parentage [40]. After removing loci fixed in Auke Creek coho salmon, 251 loci remained for analysis (electronic supplementary material, table S1). These loci showed no evidence for consistent linkage disequilibrum across years in our study population (*r*^2^ < 0.009 over all brood years).

### 2.3. Parentage assignment

We used the program FRANz [41] to assign offspring to parents by comparing the genotypes of returning adults to potential parents. This type of parentage analysis is likelihood based, meaning that the likelihood of three individuals being related in a parent–offspring triad is compared to the likelihood that they are unrelated. The laws of Mendelian inheritance and other information (e.g. age) are used to narrow down the possible parent combinations. Once the parentage likelihood is calculated, FRANz determines the maximum-likelihood pedigree. Statistical confidence in the pedigree is assessed using Markov chain Monte Carlo sampling.

FRANz runs were conducted for individual return years (2013–2019), allowing us to constrain the candidate set of parents to just the relevant brood years (i.e. 2–4 years prior; figure 3). *N*_max_ was calculated for each FRANz run by dividing the total number of potential parents by two and multiplying by 1.10 (to incorporate a 10% buffer in case some of the parents were not genotyped). The genotyping error rate used was 0.01 (the default error rate from FRANz). The maximum number of mismatching alleles allowed between dyads and triads were 5 and 7, respectively. We chose to include only individuals with 60% or more of their loci typed (150 out of 251 loci). Because of uncertainty in field-identified sex, we did not use parent sex in parentage assignment. Parent assignments were accepted if the posterior probability for parent-offspring was equal to or greater than 0.9.

We did not have known parent–offspring pairs with which to assess parentage assignment error rates. These are errors in which an individual is either falsely identified to a parent, and or not assigned to its true parent. We anticipated a small error rate for this study because almost all returning individuals each year were sampled. Sampling a large proportion of the population decreases the probability that an individual would be paired with the wrong parent. Additionally, the SNP panel had high power to detect parent–offspring pairs (greater than 100 loci with minor allele frequencies >0.25; [42]).

### 2.4. Reproductive success

We defined individual RS as the number of returning adult offspring assigned to an individual. We examined individual RS results calculated in two different ways: first by using all individuals in the parental dataset and second by using only individuals that produced at least one offspring. We defined RRS as the ratio of the mean individual RS of jacks to the mean RS of full-size males:

$$\mathrm{RRS}=\;\frac{\hat{{\mathrm{RS}}_{J}}}{\hat{{\mathrm{RS}}_{F}}}=\;\frac{(\sum_{i=1}^{N_{J}}A_{J})/N_{J}}{(\sum_{i=1}^{N_{F}}A_{F})/N_{F}},$$

where *A*_J_ and *A*_F_ are the number of offspring assigned to jack and full-size males, respectively, and *N*_J_ and *N*_F_ are the total numbers of returning jacks and full-size males for a specific brood year. This definition of RRS is commonly used in Pacific salmon literature [36,43] but differs from mating system literature that defines RRS as the RS of an individual divided by the mean RS for all individuals within a group (e.g. [44]). We determined 95% confidence intervals (CIs) for RRS estimates following [45]. We calculated RRS for each brood year separately.

We also calculated the RRS between 3-year-old jacks and 3-year-old full-size males and between 3-year-old full-size males and 4-year-old full-size males. Age refers to the total age of individuals. Comparing 3-year-old jacks and 3-year-old males allowed us to compare fish from the same brood year and maturation age but different life-histories and comparing the RRS of 3- and 4-year-old full-size males allowed us to isolate the impact of age within a single male type.

Two sources of bias in estimates of RS and RRS result from failure to assign offspring to true parents (type A error) or assigning offspring to a false parent (type B error). Both are errors in assignment, which can stem from genotyping error. As stated previously, we did not have empirical data for type A or B parentage assignment error rates. We estimated a range of bias in RRS for a range of type B error rates. The equation used was modified from Araki and Blouin's correction [46] for bias in RS between hatchery and wild fishes to account for the very low proportion of unsampled parents in our study. The unbiased RRS of jacks (RRS_UNB_) was calculated using the following equation:

$${\mathrm{RRS}}_{\mathrm{UNB}}\approx \;\frac{\hat{F_{J}}}{\hat{F_{F}}}\left(1+\frac{\eta }{\hat{F_{F}}}\right)-\frac{\eta }{\hat{F_{F}}},$$

where $\eta /\hat{F_{F}}\ll 1$, *F*_J_ and *F*_F_ are the RS of jacks and full-size males, respectively (A. J. Gharrett 2013, personal communication).

### 2.5. Immigration into Auke Creek

We examined the abundance and RS of unmarked individuals by return year and sex. Using the parentage data, we determined whether unmarked fish were probably strays (i.e. individuals that assigned with high confidence to no Auke Creek parents) or were unmarked locals (i.e. individuals that assigned with high confidence to at least one parent from Auke Creek).

## 3. Results

### 3.1. Genotyping and parentage assignment

Of the 7945 individuals sampled, 7242 (91%) were genotyped at equal to or greater than 150 out of 251 loci scored (approx. 60% of loci). The percentage of individuals genotyped at equal to or greater than 150 loci and included in further analysis each year ranged from 82% to 99% (electronic supplementary material, table S2).

Rates of parentage assignment were high. After filtering out assignments with posterior probability of less than 0.9, 78.4% of fish were assigned to two parents, 15.9% to one parent, and 5.8% to no parent. The annual percentage of individuals with zero parents assigned ranged from 3.9% to 11.4% (2014 and 2018 respectively; electronic supplementary material, table S2). One hundred and sixty-eight (66%) of the 256 individuals that were not assigned to parents had intact adipose fins, indicating that they were probably strays. Parents of unassigned fish were probably absent from our set of candidate parents (not successfully genotyped) because the posterior probabilities of the absence of an assignment were all one.

### 3.2. Reproductive success

When all potential parents were included, RS varied widely between years and sexes. The average number of offspring per individual was highest from 2009 to 2011 then dropped below replacement for both sexes from 2012 to 2015 (table 1). Females produced an average of 1.5 (s.e. = 0.1) offspring with a range from 0 to 50 offspring per individual from 2009 to 2015. Males (jacks and full-size) produced an average of 0.9 (s.e. < 0.1) offspring with a range from 0 to 36 offspring per individual from 2009 to 2015. Average annual RS of females was consistently higher than that of jacks and full-size males, except for 2015, when the female average RS was slightly less than the full-size male average. The RS of females was probably larger because males (including jacks) outnumbered females in all years (table 1).

**Table 1.** Numbers of females, jacks, and full-size males returning to Auke Creek that were successfully genotyped, sex ratio, jack frequency (of all males), and reproductive success (reported as mean (s.e.) for all returning individuals and only returning individuals that produced at least one offspring) from 2009 to 2015. Jack to full-size male relative reproductive success is shown with 95% CI.

year	*N*			sex ratio (M/F)	jack frequency	RRS (jacks:full-size)	RRS CI	RS of all individuals			RS of individuals with 1 or more offspring		
	female	male						female	male		female	male	
		jack	full-size						jack	full-size		jack	full-size
2009	161	117	116	1.55	0.46	0.34	0.27–0.43	2.8 (0.3)	0.8 (0.2)	2.3 (0.3)	5.7 (0.5)	2.8 (0.4)	4.7 (0.4)
2010	169	386	138	3.16	0.74	0.23	0.2–0.26	6.9 (0.7)	1 (0.1)	4.6 (0.6)	10.9 (0.9)	3.8 (0.3)	8.1 (0.8)
2011	254	325	189	2.1	0.64	0.25	0.22–0.29	3.9 (0.5)	0.9 (0.1)	3.5 (0.5)	11.4 (1.1)	3.6 (0.4)	8.2 (1)
2012	406	72	320	1.05	0.25	0.96	0.63–1.43	0.5 (0.1)	0.4 (0.2)	0.4 (0.1)	2.4 (0.2)	2.3 (0.8)	1.8 (0.1)
2013	329	175	273	1.34	0.39	0.53	0.39–0.7	0.8 (0.1)	0.3 (0.1)	0.6 (0.1)	2.5 (0.2)	2.1 (0.2)	2.2 (0.2)
2014	729	284	673	1.41	0.28	0.51	0.37–0.68	0.4 (<0.1)	0.2 (<0.1)	0.3 (<0.1)	1.9 (0.1)	1.4 (0.1)	1.7 (0.1)
2015	288	75	280	1.24	0.21	0.32	0.19–0.51	0.6 (0.1)	0.2 (0.1)	0.7 (0.1)	2.8 (0.3)	1.5 (0.2)	2.8 (0.3)

RS also varied between male life-history types. Jacks produced an average of 0.7 (s.e. = 0.1) offspring, while full-size males produced an average of 1.1 (s.e. = 0.1) offspring. The maximum number of offspring produced by a jack and full-size male was 21 and 36, respectively. Except for 2012, the average number of offspring produced and the variance in offspring produced by jacks was consistently less than that of full-size males (table 1). Interestingly, age 3 jacks were more abundant and had higher RS than age 2 jacks in two of the three brood years (table 2).

**Table 2.** Auke Creek coho salmon male abundance (*N*), proportion of total return (prop.), total offspring produced, mean reproductive success (mean), and variance (var.) in reproductive success for each age class from 2013 to 2015. Jacks are age classes 1.0 and 2.0.

year	age class	age	*N*	prop.	total offspring	mean	var.
2013	1.0	2	10	0.03	2	0.2	0.4
	1.1	3	54	0.14	28	0.52	0.78
	2.0	3	124	0.32	56	0.45	1.08
	2.1	4	204	0.52	134	0.66	2.19
2014	1.0	2	3	0	0	0	0
	1.1	3	196	0.21	84	0.43	0.86
	2.0	3	263	0.29	46	0.17	0.27
	2.1	4	458	0.5	145	0.32	0.62
2015	1.0	2	13	0.04	3	0.23	0.36
	1.1	3	47	0.15	37	0.79	3.3
	2.0	3	56	0.17	14	0.25	0.45
	2.1	4	204	0.64	151	0.74	3

**Figure 4.**
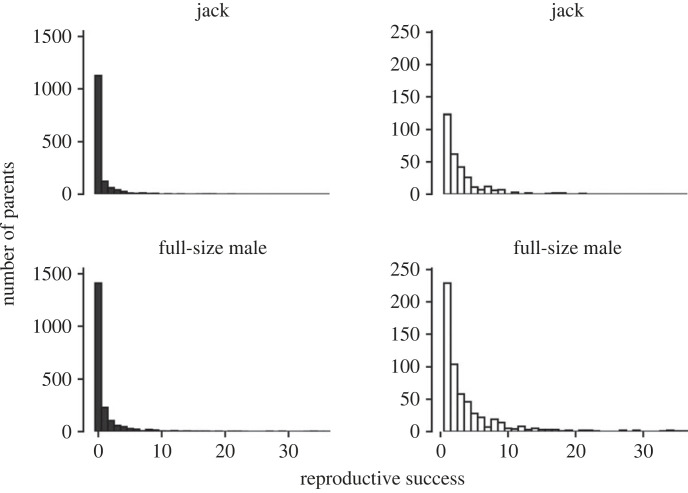
Number of Auke Creek coho salmon jacks and full-size males and their offspring from 2009 to 2015. The two panes on the left (bars with black fill) include individuals that did not produce offspring while the two figures on the right (bars with white fill) do not.

When only individuals that contributed offspring were considered (73% of returning individuals from 2009 to 2015 produced zero returning offspring from 2012 to 2019), we observed the same trend across time and between male types (figure 4). RS was highest from 2009 to 2011 before decreasing. Jacks had lower mean RS and variance in RS than full-size males, except in 2012 (table 1). Again, annual female RS was higher than jacks and full-size males, most likely owing to sex-ratio (table 2), except for one year (2015). Successful females produced an average of 5.2 (s.e. = 0.3) offspring while successful males produced an average of 3.6 (s.e. = 0.2) offspring. Of the successful males, jacks produced an average of 3.0 (s.e. = 0.2) offspring, while full-size males produced 4.0 (s.e. = 0.2) offspring.

The number of mates per female ranged from 1 to 9. Of the females that had at least one mate identified, 49.5% only had one male mate. Of the females that had more than one identified male mate, a full-size male contributed the largest number of offspring of all the male mates in 66.2% of cases. This includes all the combinations: mating with only full-size males, only jacks, or a mixture of both male types. For females that mated with at least one jack and one full-size male, a jack sired the majority of offspring in only 14.8% of the pairs.

### 3.3. Relative reproductive success

Annual RRS of jacks versus full-size males was consistently below 1, ranging from 0.23 to 0.96 (table 1). In 2012, there was a sharp increase in RRS, followed by a sharp decline for the rest of the time series. Using individuals (including those that produced zero offspring) from all years pooled together, the RRS of jacks was 0.57.

The RRS of jacks was generally larger at smaller jack frequencies except for 2015. The year 2015 had the lowest jack frequency of all the years (0.21) and a relatively low RRS (0.32) (table 1). In general, RRS was higher in years when females were more numerous (absolute abundance; table 1).

The RRS of age 3 jacks to age 3 full-size males was less than one in all three years for which we could identify the age of spawners through parentage analysis (2013–2015) and was variable across time. Across all years the RRS of age 3 jacks to age 3 full-size males was 0.52. The RRS of age 3 full-size males to age 4 full-size males was closer to 1, compared with the RRS of age 3 jacks to age 3 full-size males. The RRS of age 3 full-size males to age 4 full-size males ranged from 0.79 to 1.35. The overall value across the three years with all the individuals pooled was 1.01.

In examining a range of type B error rates, we found that if the type B error is less than 0.05 the largest per cent difference between RRS and RRS_UNB_ across focal brood years would be 2.9% (mean = 1.4%, s.d. = 1.2%). In other words, our estimates of RRS are not heavily biased.

### 3.4. Immigration into Auke Creek

The annual proportion of individuals returning with intact adipose fins (potential immigrants) ranged from 0.84% (2012) to 9.76% (2015) from 2009 to 2015, and most were females or full-size males (electronic supplementary material, table S3). Of these individuals, 63.9% were probably strays (assigned with high confidence to no parents); the remaining 36.1% assigned to at least one parent from Auke Creek. Most strays were not successful at producing offspring, but one stray (a full-size male) had 12 offspring and overall, strays produced 4.5% of all offspring (2013–2015) produced by the population (electronic supplementary material, table S4).

## 4. Discussion

Knowing the RS of individuals with different life-history traits is essential for understanding the viability of the population. Near-complete adult sampling of coho salmon at Auke Creek allowed us to successfully determine the relative reproductive success of jacks to full-size males and to evaluate the typical contribution of jacks to the next generation in a natural mating population. Although jacks were less successful on a per-individual basis, they contributed substantially to the population by fathering 23% of adults returning in 2013–2019.

### 4.1. Reproductive success of jacks

The RRS of jacks to full-size males was less than 1 in every brood year analysed. In each year except 2012, the jack group was roughly half as successful (or less) as the full-size male group. We did not find evidence that RRS was correlated with relative abundance of jacks and regular males (table 1), although we had a limited number of brood years available for comparison (seven). Despite this, we have confidence in the RRS values themselves. In every year except 2012, the 95% CI for RRS did not include 1.

Our finding of reduced RS in jacks, as measured by returning adult offspring, is consistent with research of spawning behaviour and success in salmonids. Past studies have established that competition between males of different sizes influences access to spawning females [6,21,47]. Additionally, Berejikian *et al*. [25] found that jacks, which typically entered the nest later, had lower adult-to-fry RS than full-size males. They hypothesized that when multiple males are attempting to mate, time of entering the nest determines sperm precedence and thus fertilization success. However, jacks are able to compensate for these disadvantages to some extent by having a larger gonad mass to body mass ratio than full-size males [48,49] and competitively superior sperm ([50] and references therein). Small size may provide jacks an advantage against predation and/or stranding in shallow water during their upstream migration or on the spawning grounds [51]. Jacks also spend less time at sea, so experience reduced marine mortality compared to males. To the extent that jacking is heritable [9–11], higher survival of jacks was partially accounted for in our adult-to-adult offspring measure of RS. However, to understand the persistence of a heritable trait that results in reduced reproductive success, differential mortality prior to spawning must also be accounted for [6]. The early period of marine life, shared by both jack and regular-sized males, is thought to account for less than 37% of variation in marine survival of Auke Creek coho salmon. This suggests that the additional marine mortality experienced by regular-size males would act to equalize expected lifetime fitness of these alternate male life histories, at least over the long term, thus allowing persistence of both male tactics in this population.

Auke Creek coho salmon are vulnerable to fishing mortality, primarily in the southeast Alaska troll fishery but also in the sports fishery. Both fisheries probably increase the difference in marine mortality between regular-sized and jack males. Trolling is one of the less size-selective gears used in salmon fisheries [52], but as Ricker [53] pointed out, older individuals spend more time at sea and are vulnerable over several fishing seasons, unlike jacks. Therefore, it is possible that modern fishing has increased the expected lifetime fitness of jacks, which could cause increases in jack frequencies [53]. Projecting the evolutionary consequences of fishing on jacking rates in Auke Creek coho salmon was beyond the scope of this study but could be approximated with data on heritability, exploitation rate, gear selectivity, and natural marine mortality.

Interestingly, a large proportion of jacks and full-size males were the same age. Most jacks spent two years rearing in freshwater and returned to spawn at age 3. These individuals returned at the same age as the full-size males of their cohort who spent one year in freshwater and one year in saltwater. There were very few jacks that returned to spawn at age 2. Age 3 jacks had lower RS than age 3 full-size males while age 3 full-size males had comparable RS to age 4 full-size males, indicating that RS was more influenced by male type than age, which underscores the role of male body size in reproductive success in coho salmon.

### 4.2. Immigration into Auke Creek

The weir and tagging regime implemented at Auke Creek provided a rare opportunity to address reproductive success of immigrant coho salmon. Most fish identified as likely strays were not successful at producing offspring; yet overall, they were responsible for approximately 4.5% of all offspring produced from 2013 to 2015 even though they averaged less than 4% of spawning adults during those years. These data imply minimal selection against immigrant individuals, but it would be helpful to confirm the source populations of the strays and to compare the RS of true stray and Auke Creek individuals once more data become available. Interestingly, very few jacks with intact adipose fins returned to Auke Creek between 2009 and 2015. This observation aligns with other studies on coho salmon [54] and chinook salmon [55–57] that demonstrate younger age classes tend to stray less than older age classes. Although jacks tended to be the same total age as full-size males in our study, they spent more time in freshwater and less time in the ocean. This could lead to stronger olfactory imprinting and does result in less time between imprinting and recall, but the evidence for these factors affecting straying is equivocal [58]. Continued genetic monitoring of this population could provide more information about the life history and reproductive success of coho salmon straying into Auke Creek.

### 4.3. Implications for management and conservation

Jacks are commonly undersampled in studies of wild populations because their small size makes them difficult to enumerate (e.g. at counting towers, temporary weirs, and in aerial surveys), they are often assumed to have a small contribution to the next generation, and they are considered unimportant in commercial and recreational fisheries. This study shows that jacks can have a large contribution to RS of returning adult offspring, and that ignoring jacks can drastically influence what conclusions can be drawn from the RS results. For example, consider a scenario in which jacks contribute a significant number of offspring each year but are left out of the counts of returning spawners. The number of fish returning would be attributed to the full-size adult counts in prior years and this would overestimate individual RS and affect projections of population productivity.

Jacks are also relevant to studies that examine the relative RS of hatchery versus natural-origin salmon. The general trend is that the RS of hatchery-origin fishes is lower than that of natural-origin fishes [36,43], but it depends on the type of hatchery (integrated or segregated) and factors like the source of broodstock and how long fish are held in the hatchery prior to release [59]. Koch & Narum [36] suggested that jacks should be accounted for in RS calculations. They found in some studies when jacks were analysed separately from full-size males (which some studies do not do), there appeared to be a smaller difference in the RS of natural- and hatchery-origin individuals when spawning naturally (this was true for both males and females). Overall, including jacks in studies is advisable, because jacks and full-size males may have different mean RS and variance in RS and may represent a substantial portion of the population. Shaul *et al*. [60] found that coho salmon jacks made up an average of 44% of the male escapement in Auke Creek and less than 0.5% in the nearby Berners River, suggesting that the effect of excluding jacks when considering RS and population productivity could vary widely even within a single geographical region. Coho salmon populations in Oregon [61] and sockeye salmon populations in Bristol Bay [51] demonstrate similar evidence of variation in jack frequencies among populations.

Our study is relevant to the question of whether, and at what proportion, to include jacks in hatchery broodstock. In hatcheries that attempt to mimic natural conditions to minimize divergence from wild stocks, efforts should be made to investigate whether including jack fathers at a rate comparable to that in wild populations would be beneficial. In hatcheries that practice random mating of full-size males and females but exclude jacks from their broodstock, their random mating is not truly random. In this case, the current hatchery mating structure of excluding jacks is not representative of the natural mating structure of Auke Creek coho salmon, but RRS of less than 1 in our study suggests that including jacks in proportion to their abundance in returning adults also does not approximate natural conditions. Including jacks may be less crucial in other populations that have fewer jacks or jacks that are less successful. On the other hand, it could be more important in populations where there is naturally a large jack contribution.

When considering adding jacks to broodstock, it is important to acknowledge a complicating factor, specifically how hatchery rearing can affect the resulting broodstock. The proportions of each age class and their subsequent impact on the following generation in a natural population is most likely different from the impact that those same proportions would have in a hatchery setting. Hatchery rearing conditions such as high feed quality and optimal water temperatures increase juvenile growth rate, which influences the probability of early maturation [19,62–66]. This can result in a higher proportion of jacks than that of the natural population of the broodstock. This higher-than-natural proportion of jacks conflicts with the goal of integrated hatcheries, which is to not diverge from natural population structure, and conflicts with segregated hatcheries, which want to produce full-size fish for harvest. Because there is a component to jacking that is heritable, actively adding jacks into broodstock may only exacerbate this divergence. A study by Larsen *et al*. [20] on chinook salmon found that while integrated hatcheries slow the rate of genetic change in the threshold for early male maturation, they produce a large proportion of males that mature early. This phenomenon makes it difficult to strike a balance between incorporating jacks without adding to the problem of hatchery reduction of age at maturity and overshooting the proportion of jacks in the natural population. Both topics merit further consideration. Ultimately, the decision to include jacks in breeding programmes requires studying hatchery crosses and population-specific information and depends on the goals of the hatchery programme and many other factors.

Jacking can be a productive life-history tactic and it should not be neglected in studies of population and evolutionary dynamics of Pacific salmon. Conservation programmes, including hatcheries, would benefit from continued research on the contributions of jacks in natural populations.

## Data Availability

All the supporting data and code are published as electronic supplementary material [67]. R software: https://www.R-project.org/. FRANz software: https://www.bioinf.uni-leipzig.de/Software/FRANz/About.html.
